# Editorial: Authenticity of Probiotic Foods and Dietary Supplements

**DOI:** 10.3389/fmicb.2021.789049

**Published:** 2021-11-29

**Authors:** Vincenzina Fusco, Francesca Fanelli, Evandro Leite de Souza

**Affiliations:** ^1^National Research Council of Italy - Institute of Sciences of Food Production (CNR-ISPA), Bari, Italy; ^2^Federal University of Paraíba, João Pessoa, Brazil

**Keywords:** probiotic food, probiotic supplement, authenticity, legislation, viable but not culturable

Probiotics are viable microorganisms, which, if ingested in adequate amounts, confer a health benefit to the host (Hill et al., 2014). An authentic probiotic food must contain the number of viable cells of the specific probiotic strain correctly cited on the label and provide the claimed beneficial health effects, which should not be deceptive for consumers (Di Lena et al., 2015).

While the production and the global market of probiotic foods and supplements is increasing worldwide, the indication of the probiotic microorganisms reported on the label might be misleading both at quali- and quantitative levels. Indeed, several studies have reported inconsistency between the actual content of probiotics in commercial foods and dietary supplements and their label information in terms of the dose of viable cells and type of microorganism (at genus, species, or strain level) (Fusco et al., 2021). This scenario is further complicated by the taxonomic amendments that have occurred in the last years mainly due to the availability of complete genomes of (probiotic) strains (Makarova et al., 2006; Briczinski et al., 2009; Loquasto et al., 2013; Holzapfel and Wood, 2014; Milani et al., 2014; Lugli et al., 2019; Zheng et al., 2020). As an example, the *Lactobacillus* genus has been recently reclassified into 25 genera including the amended genera *Lactobacillus, Paralactobacillus*, and 23 novel genera (Zheng et al., 2020). The scientific community as well as regulators and consumers must deal with these taxonomic revisions as soon as they occur.

All the above findings prompt the need to improve and standardize the methods to assess the authenticity of the probiotic foods and supplements and harmonize their regulation at the global level.

The most valuable methods are those able to distinguish among dead, viable, and viable but not cultivable (VBNC) cells, which might be present in probiotic foods due to the biotic and abiotic stresses that probiotics undergo during the production, storage, distribution, and consumption (Fusco and Quero, 2014; Fiocco et al., 2020; Fiore et al., 2020; Fusco et al., 2021). Nevertheless, in recent years, alternative methods including fluorescent *in situ* hybridization (FISH) (Babot et al., 2011), flow cytometry (Wilkinson, 2018), or combination of multi-omics approaches, such as the promising propidium monoazide (PMA)-metagenomics (Fusco et al., 2021) and culturomics, have been proposed.

This Research Topic aims to collect the latest research on the authenticity evaluation of probiotic foods and dietary supplements. It covers a total of six articles, including three original researches, two methods, and one review, with a focus on the legislation, assessment, development, and application of chemical, molecular, and omics methods to evaluate the authenticity of probiotic foods and supplements.

Pammi et al. presented the characterization of a traditional Indian rural drink obtained by the Toddy Palm Nectar, indicating its probiotic potential by nutritional profiling and isolation of lactic acid bacterial strains, which could be exploited in developing therapeutic applications.

Lorbeg et al. evaluated the quality of dietary supplements containing viable bacteria available in Slovenian pharmacies using plate counting, matrix-assisted laser desorption ionization time-of-flight mass spectrometry (MALDI-TOF MS), and species- or subspecies-specific PCR. Besides revealing mislabeling of *Lacticaseibacillus casei* in some products, the study confirmed that MALDI-TOF MS can be effectively used in the quality control of probiotic products, being as a faster and simpler alternative to PCR identification. It was also indicated that the generation of a dedicated in-house library may further improve the identification accuracy at the species and sub-species level.

Colom et al. performed a clinical trial to directly investigate, for the first time, the presence and germination of the probiotic strain *Bacillus subtilis* DE111® after the ingestion of commercially available capsules in the small intestine using a novel methodology involving healthy adults with an ileostomy. *B. subtilis* DE111® spores were able to retain their viability during the transit through the stomach and germinate in the small intestine of humans within 3 h of ingestion.

Deidda et al. described the first investigation of bifidobacterial strain typing using Fourier transform infrared (FTIR) spectroscopy. Compared to pulsed-field gel electrophoresis (PFGE), whole-genome sequencing (WGS), multilocus sequence typing (MLST), FTIR resulted more informative and able to differentiate strains within the *B. animalis* subsp. *lactis* group.

Weitzel et al. illustrated how the implementation of the analytical procedure lifecycle management (APLM) in plate counting can lead to lower variability and significantly impact the manufacturing process, reduce costs for industries and improve the quality evaluation of probiotic products, while supporting claims of dose and, therefore, health benefits.

A very comprehensive review by Mazzantini et al. described the many incongruences in the compositional quality of some probiotic formulations available on the worldwide market, highlighting the need of using recommended, standardized, and updated methodologies for analyzing and labeling probiotic products.

All the contributions presented in this topic confirm the need to urgently harmonize the regulation on probiotics worldwide, as promoted by the International Scientific Association for Probiotics and Prebiotics (ISAPP, https://isappscience.org) and the International Probiotics Association (IPA, https://internationalprobiotics.org), to develop updated, standardized, faster, and reliable methods to assess the authenticity of probiotics and ensure the criteria of taxonomy, viability, stability, and safety needed to characterize probiotic foods and supplements. These advances are expected to generate improvements in the manufacturing process and quality control to guarantee the development and validation of probiotic-based therapeutical strategies, as well as in the defense of the consumers' right of being correctly informed and aware of their choices.

## Author Contributions

All authors write, revised, and accepted the manuscript.

## Conflict of Interest

The authors declare that the research was conducted in the absence of any commercial or financial relationships that could be construed as a potential conflict of interest.

## Publisher's Note

All claims expressed in this article are solely those of the authors and do not necessarily represent those of their affiliated organizations, or those of the publisher, the editors and the reviewers. Any product that may be evaluated in this article, or claim that may be made by its manufacturer, is not guaranteed or endorsed by the publisher.
